# Illinois State Dental Society

**Published:** 1868-03

**Authors:** 


### ILLINOIS STATE DENTAL SOCIETY.

G. H. Cushing, of Chicago, President; A. W. French, of Springfield, Vice President; M. S. Dean, of Chicago, Secretary; J. N. Crouse, of Mt. Carroll, Treasurer; W. W. Allport, of Chicago, Librarian.

Executive Committee; A. W. French, of Springfield; E. H. Kilbourne of Aurora; H. N. Lewis, of Quincy; S. Babcock, of Springfield; L. F. Abbott, of Wilmington.

Fifth regular meeting will be held at Springfield on the second Tuesday (12th) of May, 1868, at 10 o'clock A. M.

At the last meeting of the Illinois State Dental Society, Drs. A. W. French and H. J. Smith were appointed a special committee to furnish subjects for discussion at the next meeting.

In the performance of this duty the undersigned, after mature deliberation, have the pleasure of presenting the following topics for the consideration of the Society:

Operative Dentistry: Treatment of decayed six year Molars. Plugging Pulp Cavities and Canals. Receding of the Gums in persons of middle age, Cause and Treatment. Diseases of the Gums. Observed effects of the premature extraction of the Temporary Teeth. Facial Neuralgia.

Anæsthesia: Local; General.

Mechanical Dentistry: Vulcanized Rubber; Gold Plates; Continuous Gum. New Materials.

Miscellaneous: Essays or prepared addresses upon these or any other subject pertaining to the profession would, no doubt, be gladly received by the Society, and would add much to the interest of the meeting.

A. W. FRENCH, }

H. J. SMITH, } Committee.

